# Meta-adaptation in the auditory midbrain under cortical influence

**DOI:** 10.1038/ncomms13442

**Published:** 2016-11-24

**Authors:** Benjamin L. Robinson, Nicol S. Harper, David McAlpine

**Affiliations:** 1 University College London Ear Institute, 332 Gray's Inn Road, London WC1X 8EE, UK; 2 Southwark and Central Integrated Psychological Therapies Team, The Maudsley Hospital, South London and Maudsley NHS Foundation Trust, Denmark Hill, London SE5 8AZ, UK; 3 Department of Physiology, Anatomy, and Genetics, University of Oxford, South Parks Road, Oxford OX1 3QX, UK; 4 Department of Engineering Science, Institute of Biomedical Engineering, University of Oxford, Old Road Campus Research Building, Headington, Oxford OX3 7DQ, UK; 5 The Australian Hearing Hub, Macquarie University, 16 University Avenue, Sydney, NSW 2109, Australia

## Abstract

Neural adaptation is central to sensation. Neurons in auditory midbrain, for example, rapidly adapt their firing rates to enhance coding precision of common sound intensities. However, it remains unknown whether this adaptation is fixed, or dynamic and dependent on experience. Here, using guinea pigs as animal models, we report that adaptation accelerates when an environment is re-encountered—in response to a sound environment that repeatedly switches between quiet and loud, midbrain neurons accrue experience to find an efficient code more rapidly. This phenomenon, which we term meta-adaptation, suggests a top–down influence on the midbrain. To test this, we inactivate auditory cortex and find acceleration of adaptation with experience is attenuated, indicating a role for cortex—and its little-understood projections to the midbrain—in modulating meta-adaptation. Given the prevalence of adaptation across organisms and senses, meta-adaptation might be similarly common, with extensive implications for understanding how neurons encode the rapidly changing environments of the real world.

## Abstract (web summary)

Neurons in the auditory midbrain are known to modify their firing rates in response to changes in sound intensity. Here the authors show that in guinea pigs, such modifications occur faster when neurons re-encounter the same environment, a phenomenon they term meta-adaptation.

## Graphical abstract

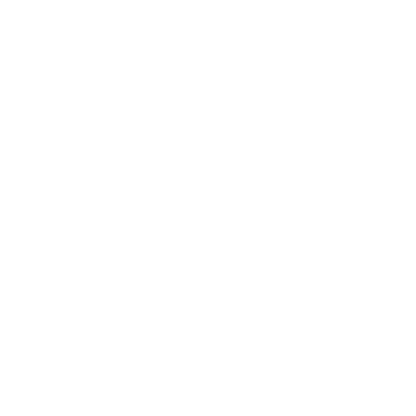

To represent the world's vast range of sounds, sights and other sensations, neurons adapt their sensitivity to accommodate current environmental statistics (such as overall intensity or contrast), enabling them to code auditory, visual and tactile objects with greater efficiency or precision^1^,^2^,^3^,^4^,^5^,^6^,^7^,^8^,^9^,^10^,^11^,^12^,^13^,^14^,^15^. However, it takes time for sensory systems to assess these statistics with precision and adapt to them (approximately half a second in audition^16^), during which the neural representation, and the organism's survival, are potentially compromised. Given the fundamental trade-off between adapting rapidly and adapting precisely, we investigated whether sensory systems can exploit prior knowledge of an environment to mitigate this conflict.

Employing a stimulus that alternates between loud and quiet environments, we show that neurons in the auditory midbrain adapt more rapidly each time the loud environment is re-encountered, eventually doubling the speed at which they adapt to it. This phenomenon, which we term meta-adaptation, suggests that prior knowledge of an environment enables neurons to assess features of that environment more rapidly. The existence of such sensory memory is surprising; neurons in the auditory midbrain are thought to retain sensory information over just fractions of a second, two orders of magnitude shorter than the learning effect we describe^16^. Given the extensive corticofugal pathways between cortex and midbrain—the functions of which are a matter of intense research interest^17^,^18^,^19^,^20^,^21^,^22^,^23^,^24^,^25^—we assessed whether these pathways might play a role in the generation of meta-adaptation in the midbrain. By reversibly inactivating auditory cortex, using a well-described cryoloop cooling method^26^,^27^, we find that acceleration of adaptation is attenuated. We hypothesize that the meta-adaptation—the acceleration of adaptation with experience—employs a cortical memory trace of past environments to maximize the speed with which midbrain neurons adapt to a change in the environment.

## Results

### The switching stimulus

**Figure 1:**
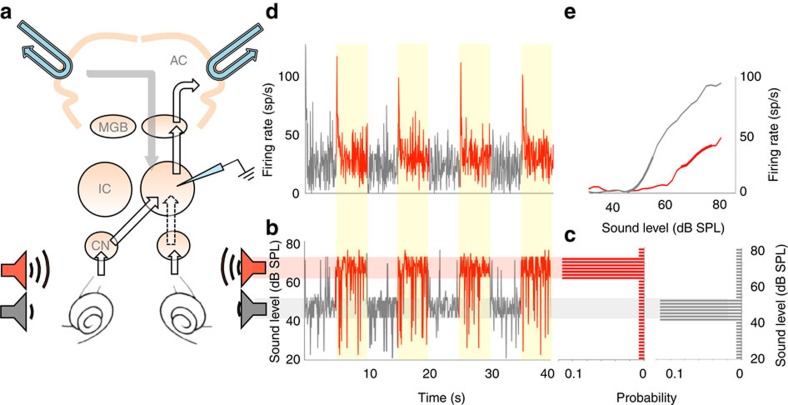
Auditory midbrain responses to switches between sound-intensity environments. (**a**) Recording set-up. Electrode in IC. IC receives input from ears via contralateral cochlear nucleus (CN), and, indirectly, ipsilateral CN (dotted line). Auditory cortex (AC) receives from IC via medial geniculate body (MGB) and also gives bilateral feedback. Cooling loops (blue). (**b**) Section of broadband noise sound stimulus, quiet (grey) and loud (red) environments. (**c**) Level distribution per environment type. (**d**) Firing rate over time of single neuron to stimulus. (**e**) Firing rate versus stimulus intensity of neuron per environment. Superimposed thick lines represent high-probability regions of **c**.

We first ascertained whether adaptive coding—the process by which neural coding becomes suited to the stimulus statistics—is itself subject to adaptation. To do so, we assessed the speed at which neurons in the auditory midbrain (inferior colliculus (IC); Fig. 1a) adapt with increasing experience of a sound environment. We generated two different environments, loud and quiet, characterized by the most common (80%) sound intensities spanning the ranges 69–81 or 45–57 dB SPL (decibels sound pressure level), respectively. Each sound environment comprised a continuous broadband noise whose intensity was randomly selected every 50 ms from that environment's distribution of sound intensities. The two environments alternated every 5 s (Fig. 1b,c), to create a switching stimulus^1^, in which each environment was presented on at least 24 occasions. This was presented diotically (identically to both ears, via calibrated headphones) to anaesthetized guinea pigs and the responses of single neurons in the IC were recorded using extracellular tungsten microelectrodes (see the ‘Methods' section). 78 neurons were recorded from 14 guinea pigs.

### Adaptation accelerates with increasing stimulus exposure

In previous studies, using the same switching stimulus, we demonstrated^1^,^2^ that most IC neurons (∼98%) respond to a transition to the loud environment by adapting their firing rates (Fig. 1d) to accommodate the most commonly occurring sound intensities. Specifically, we showed that switching from a quiet to a loud environment elicits an exponential decay in a neuron's firing rate—from an initially high rate to a lower, steady-state rate (∼160 ms median time-constant, 74–204-ms interquartile range). This adaptation of spike rate—associated with a shift of neural firing-rate-versus-sound intensity (rate-intensity) functions (Fig. 1e)—tended to position a neuron's most intensity-sensitive domain (typically the slope just above threshold), over the most commonly occurring intensities^1^,^2^. At a population level, this form of adaptation improves the coding precision of the most common sound intensities^2^.

**Figure 2:**
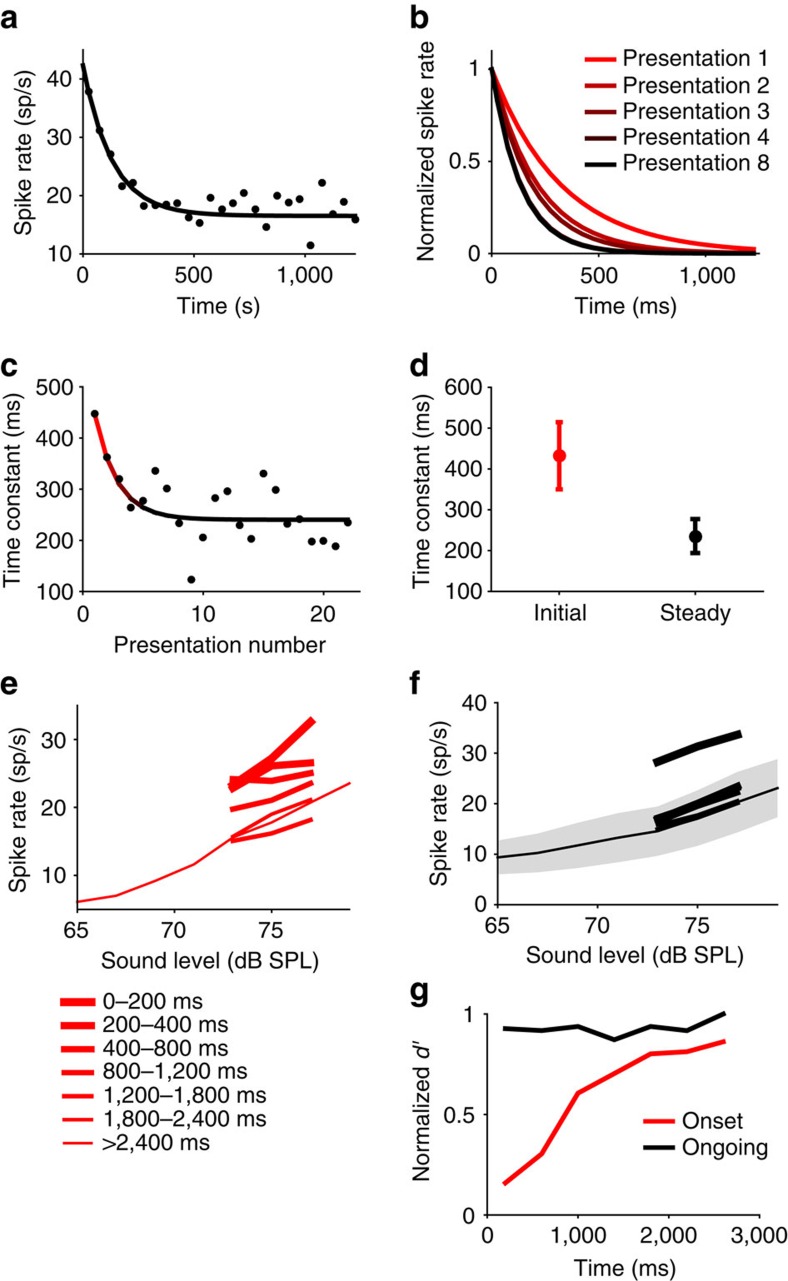
Meta-adaptation in the auditory midbrain. All plots for loud environment. (**a**) Average firing-rate over time of a neural population of 78 IC neurons (from 14 guinea pigs), averaged over presentations 11–24. Line, exponential fit *τ*_adapt_=337 ms (exponential versus flat line, *P*<<0.0001, *f*-test). (**b**) Exponential fits after different numbers of presentations (exponential versus flat line; *P*<0.05 in all 5 cases, *f*-test). (**c**) Adaptation time-constant versus presentation number. Line, exponential fit *τ*_meta_=1.9 presentations (exponential versus flat line; *P*=0.013, *f*-test). (**d**) Bootstrapped initial (red, median *τ*_adapt_=432 ms over 10,000 bootstraps) and steady state (black, median *τ*_adapt_=234 ms) adaptation time-constants, 95% confidence intervals. (**e**) For the same population of 78 neurons the average rate-intensity functions for the high-probability sound levels at times from environment onset, before meta-adaptation. A five-point average was used for display. (**f**) As **e** but after meta-adaptation. The grey shaded region indicates the standard deviation in the population response to a given level. (**g**) The neural population coding precision (*d*′) versus time since environment onset, before (red), after (black) meta-adaptation. All *d*′ normalized to the maximum of the black line.

Here, we assessed the neural population response to the loud environment, defined as the average firing rate over the recorded neural population in each 50-ms epoch since the start of the loud environment, averaged over a number of presentations (adaptation to the quiet environment was less pronounced^1^ and is excluded from our analysis). Adaptation, in the form of a decay in population firing-rate over time, is clearly evident in the population response (Fig. 2a; 78 neurons, averaged over presentations 11–24), and can be quantified in terms of an exponential decay with a time-constant (*τ*_adapt_) of 337 ms (exponential versus flat line, *P*<<0.0001, *f*-test). This is slightly larger than, but of a similar order of magnitude to, the time-constants we previously reported for individual IC neurons^1^. To determine whether adaptive coding is influenced by experience of the switching stimulus, we calculated the time-constant of adaptation (of the population response) for successive 5-s exposures to the loud environment (Fig. 2b, exponential versus flat line; *P*<0.05 in all 5 cases, *f*-test). Unexpectedly, we found that adaptation accelerates with increasing presentations of the environment (Fig. 2c). This increase in the rate of adaptation can itself be characterized by an exponential (Fig. 2c, *τ*_meta_=1.9 presentations of the environment, exponential versus flat line; *P*=0.013, *f*-test), and indicates that the speed of adaptation of the population response increases by a factor of ∼2, from 432 to 234 ms (Fig. 2d). We term this higher-order adaptation ‘meta-adaptation'.

Meta-adaptation is inherently more challenging to measure for individual neurons, since it ideally requires adaptation time-constants to be assessed on a single-presentation basis (that is, in response to individual exposures to the 5-s stimulus). Since neural responses are intrinsically noisy, this limits the accuracy with which these measurements can be made from single presentations, whilst averaging responses of individual neurons over many presentations, rather than over the neural population to one or a few presentations, abolishes the temporal resolution required to observe the effect. Despite this limitation, however, responses of a proportion of neurons (15%; 12/78 neurons) were robust enough to observe statistically significant meta-adaptation, measured using the same methods as for the population (*τ*_meta_=0.4–6.2, exponential versus flat line; *P*<0.05, *f*-test; Supplementary Fig. 1a,b). Further, removing these neurons from the larger sample of neurons had little effect on the meta-adaptation observed in the remaining population response (Supplementary Fig. 1c); meta-adaptation was significantly present (*τ*_meta_=1.9 presentations, exponential versus flat line; *P*=0.04, *f*-test), and of only marginally less strength (*τ*_adapt_ increased in speed from 404 to 234 ms with repeated exposures to the loud environment). Thus, meta-adaptation is evident at the level of single neurons, and in the population response, even when the population response is analysed in the absence of the most-robustly responding neurons in which it was evident on a single-neuron basis. Finally, by examining the time-constant of adaptation for each of the 78 neurons for every consecutive presentation of the loud environment (Supplementary Fig. 1d), we find that the mean time-constant over the neurons decreases as the number of presentations increases, showing statistically significant decay (*τ*_meta_=1.2 presentations, exponential versus flat line; *P*=0.0067, *f*-test).

### Persistence and possible function of meta-adaptation

How persistent is meta-adaptation? Do neurons recover from their meta-adapted state sometime after the end of the switching stimulus, or is meta-adaptation an enduring change in brain state? To answer this question, we took advantage of the fact that we made successive recordings (from different neurons) in each animal. If meta-adaptation reflects an enduring change in brain state, occurring once only for each animal in response to our stimulus, we would expect to observe it in only the very first recording. We therefore re-analysed our data excluding data from the very first neural recording to the switching stimulus in each animal—if neurons recover from meta-adaptation, acceleration of adaptation should still be apparent over the time course of the responses to the remaining presentations of the switching stimulus. Meta-adaptation was clearly evident in the neural population response of the remaining 64 later-recorded neurons (*τ*_meta_=2.2 presentations of the environment, exponential versus flat line, *P*=0.012, *f*-test), with adaptation time-constants decreasing from 426 to 235 ms over the course of the switching stimulus. Hence, meta-adaptation recovered in the interval between recordings (which were in the order of 5–20 min), and therefore likely represents a form of sensory memory that fades over time, rather than a persistent change in brain state.

What is the functional relevance, if any, of an accelerating rate of adaptation? Does it accelerate the adjustment of rate-intensity functions to improve coding precision of common sound levels? To address this question, we examined the change in the average rate-intensity function of the neural population (78 neurons) over the 5-s time course of the loud environment, before and after meta-adaptation was complete (for highly probable intensities only; see the Methods section). Before meta-adaptation, the population rate-intensity function did not adapt to its settled state until at least 1,200 ms following the switch to the loud environment (Fig. 2e). Once meta-adaptation was complete, however, the rate-intensity function largely settled to its final configuration some 200 ms following the switch (Fig. 2f). This dependence of the rate at which rate-intensity functions settled to their final, adapted state was also apparent at the single-neuron level, in the 12 neurons for which meta-adaptation could be measured from single-trial responses (Supplementary Fig. 2a–d), and in the population response (66 neurons) when these 12 neurons were removed from the analysis (Supplementary Fig. 2e,f).

We quantified the functional effects of meta-adaptation by calculating the *d*′ (a measure of coding precision^28^,^29^,^30^,^31^,^32^) of the population (78 neurons) rate-intensity function for the highly probable intensities as a function of time (Fig. 2g), corroborating the change we observed in the rate-intensity functions. The *d*′ measure is high when the slope of the function is steep, consistent intuitively with a smaller change in the coded variable eliciting a larger change in the firing rate. Likewise, *d*′ is smaller when the variability is high (see the ‘Methods' section). Whilst coding precision before meta-adaptation clearly increases over the first 1,500 ms or so of the 5-s exposure, the adaptation of coding precision following meta-adaptation occurs so rapidly that it cannot be fully captured by our measure (that is, within ∼300 ms). This suggests that meta-adaptation enables the brain to converge rapidly to a more-precise neural representation of the most common sound intensities. Further, coding precision during the steady state (>1,500 ms) is slightly higher following meta-adaptation than before, largely due to the relatively steeper population rate-intensity function. Thus, before meta-adaptation occurs, neural firing rates, rate-intensity functions and coding precision all appear to adapt slowly, whereas after meta-adaptation they adapt quickly.

### Cooling cortex attenuates meta-adaptation

The relatively long time course of meta-adaptation in the IC is somewhat surprising, given the relatively short time course over which IC neurons are thought to integrate information^1^,^16^. We hypothesized, therefore, that meta-adaptation in the auditory midbrain might be mediated by cortical feedback. Corticofugal pathways are extensive in humans, and other mammals, and are thought to play a role in adjusting neural sensitivity and plasticity of receptive fields, although their exact function in hearing and other senses remains to be determined^17^,^18^,^19^,^20^,^21^,^22^,^23^,^24^,^25^. To assess the possible role of feedback from both cortices, we inactivated auditory cortex bilaterally by means of localized cooling (using a cryoloop), whilst simultaneously recording responses of IC neurons (52 neurons) to the switching stimulus. Before applying our localized cooling method to this question, we performed extensive calibration experiments, measuring the temperature at different depths from the cortical surface with thin insulated-thermocouple probes (Supplementary Fig. 3a,b), and using electrodes placed either within auditory cortex itself (Supplementary Fig. 3c), or just inferior to cortex (Supplementary Fig. 3d), to ensure that our cooling was sufficient to inactivate auditory cortex, whilst avoiding inactivating subcortical structures by direct cooling (see the ‘Methods' section). Neural responses after cessation of cooling were seen to recover in a subset of midbrain neurons which could be held for long enough after cessation of cooling (Supplementary Fig. 4).

**Figure 3:**
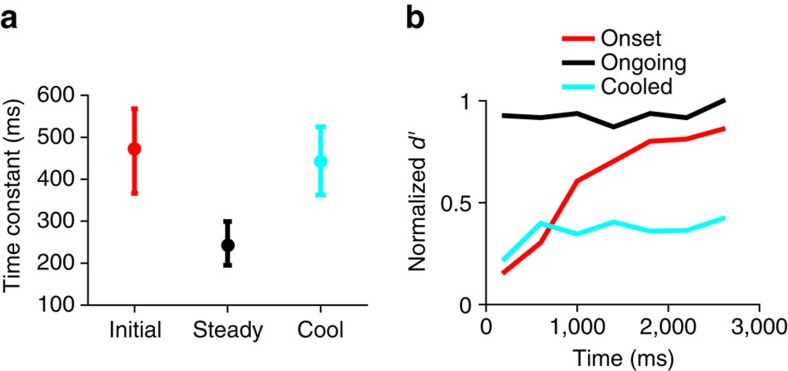
The effect of cooling the auditory cortex on midbrain adaptation. All plots for loud environment. (**a**) For average population response of the 52 of the 78 IC neurons for which cortical cooling was performed, the bootstrapped initial (red, median *τ*_adapt_=472 ms over 10,000 bootstraps) and steady state (black, median *τ*_adapt_=243 ms) time-constants of adaptation before cooling, and bootstrapped time-constant of adaptation after cooling (blue, median *τ*_adapt_=443 ms). (**b**) Neural population coding precision (*d*′) versus time since environment onset, before (red), and after (black) meta-adaptation, and after cooling (blue), 95% confidence intervals. The blue line is calculated from the population rate-intensity function of the same 52 neurons in **a**, the red and black lines used all 78 neurons. All *d*′ normalized to the maximum of the black line.

Before cortical cooling, meta-adaptation was present (exponential versus flat line *P*=0.0092, *f*-test) in the 52 neuron population response, and to a similar degree (*τ*_adapt_ decreased from 472 to 243 ms) to that observed for the larger population of IC neurons (78 neurons). Surprisingly, cooling auditory cortex substantially increased the time-constant of adaptation in IC neurons from 243 to 443 ms, a value statistically indistinguishable (by bootstrap confidence intervals) from the 472 ms observed before meta-adaptation had occurred in this population (Fig. 3a), suggesting that cortical cooling attenuates meta-adaptation. To confirm that cortical cooling inhibits the generation of meta-adaptation, we initiated the switching stimulus in the cooled state for 29 of the 52 neurons. In contrast to when the cortex is active, the time-constant of adaptation during cortical cooling does not significantly change with repeated exposure to the loud environment (exponential versus a flat line; *P*=0.054, *f*-test), this despite meta-adaptation being present before cooling for the same population of 29 neurons (exponential versus flat line, *P*=5.4 × 10^−4^, *f*-test). The non-significant fit of the exponential function in the cool state (Supplementary Fig. 5) was also slower (*τ*_meta_ 3.7 versus 3.0 presentations) and shallower (47 versus 35% drop to steady state) than in the warm state for the same 29 neurons. This does not preclude the possibility that some meta-adaptation, of a degree undetectable by our particular tests, might be generated intrinsically within the IC itself, or from some other input.

We also examined the effect of cortical cooling on how quickly coding precision adapts during the loud environment (Fig. 3b, 52 neurons for cool, 78 neurons for warm, rescaled appropriately), using the standard *d′* measure^28^,^29^,^30^,^31^,^32^ on the population rate-intensity function for the most common sound intensities only. Coding precision in the cooled state improves slightly over the first 600 ms of the stimulus (Fig. 3b, blue line), more slowly than in the warm meta-adapted state (black line), but faster than before meta-adaptation (red line). However, coding precision in the cooled state remains only marginally more precise than before any adaptation has occurred in the warm, pre-meta-adaptation state (start of black line). This suggests that cooling the cortex not only attenuates meta-adaptation, but also limits the capacity of adaptive phenomena to improve the precision of neural coding.

### Models of estimation of the mean sound intensity

**Figure 4:**
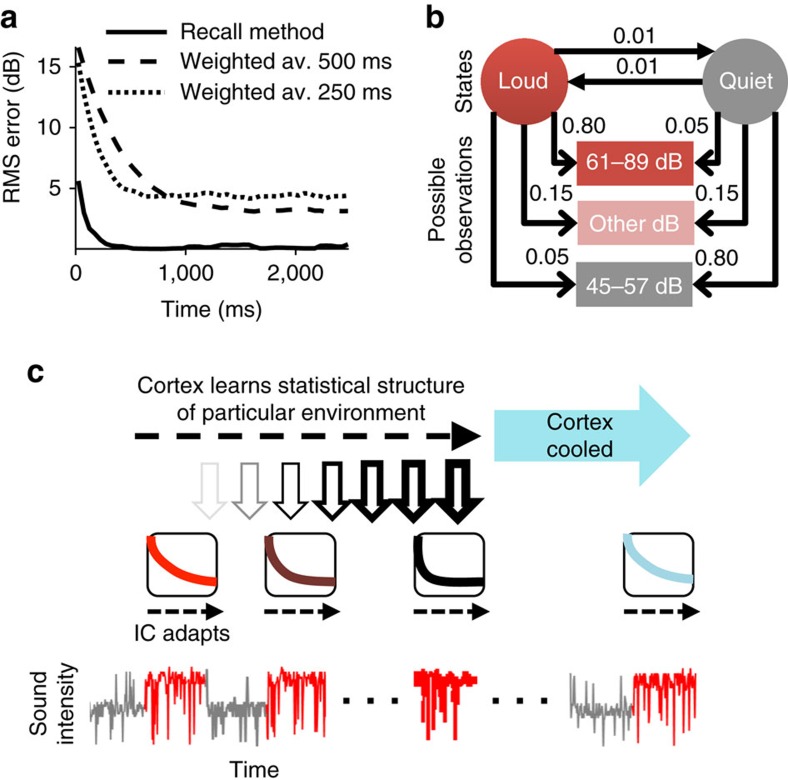
Suggested functional implications of adaptation to the mean sound level. (**a**) RMS error of estimation of the mean level of the loud environment, as a function of time since the switch to the loud environment. The weighted-average method of estimation: averaging with an exponential with time-constant 500 ms (dashed line), and 250 ms (dotted line). The recall method of estimation: not averaging but recognizing a previously experienced environment (solid line, modelled using a hidden Markov model). (**b**) Hidden Markov model of switching stimulus. States (circles), possible observations (rectangles), transition probabilities (solid head arrows) and output probabilities (open head arrows). (**c**) Schematic of the overall hypothesis. When first encountering an environment a simple weighted average method is used to estimate and adapt out the mean sound level; however with experience of the environment, its mean can simply be recalled by the cortex, a faster method that allows for faster adaptation. Cooling the cortex attenuates this phenomenon.

Our data suggest that adaptation in the auditory midbrain is not a static phenomenon but, instead, accelerates with experience, and that this acceleration of adaptation is reduced or absent in the absence of feedback from auditory cortex. To understand the implications of these findings, we posit a model that accounts for the data. We hypothesize two distinct functional methods by which the brain might rapidly estimate the overall mean sound intensity of an environment (that is, its mean intensity over a long duration, such as seconds) to adapt to it, and hence to reach a state of more precise coding. The first of these two methods, the ‘weighted-average method', rapidly estimates the overall mean as the exponentially decaying, weighted-average of the intensities over the very recent past (a ‘sample mean')^1^,^33^. Although this method requires no learning about particular previous environments, the precision of the estimate can be relatively poor. Consider estimation of the mean of the loud environment of the switching stimulus following a switch from the quiet environment (being statistically stationary, each environment has a particular fixed overall mean). For a weighted average with a time-constant of 500 ms, such as before meta-adaptation, the estimate of the overall mean of an environment varies substantially depending on the recent history. The root-mean-square (RMS) error of the estimated mean relative to the true overall mean settles at ∼3 dB about 1 s into the switch period (Fig. 4a, see the ‘Methods' section). A faster time-constant of 250 ms, such as we observed after meta-adaptation is complete, enables more rapid estimation of the mean, but shows even less accuracy (∼5 dB RMS error), and would render adaptive coding ineffective: deviations of just twice the RMS error magnitude encompass the entire neural dynamic range for sound intensity of most IC neurons.

A more rapid and precise method, however, by which the overall mean intensity might be estimated would be to incorporate prior knowledge about different environments: to recognize when an environment is re-encountered and then recall its overall mean intensity. For our switching stimulus, with its quiet and loud environments—each with a distinct overall mean—the task then becomes one of deciding which environment is currently experienced, given the past few sound intensities. We term this method the ‘recall method', and it can be functionally (black box) modelled in terms of a hidden Markov model^25^ employed to recognize rapidly the remembered environments, given the recent sound input (Fig. 4b; see the ‘Methods' section). The overall mean intensity is then estimated by weighting the remembered overall mean intensity for each environment by the posterior probability that either environment is currently encountered. By this method, the overall mean intensity is rapidly and precisely assessed, as compared with the apparently simpler, but ultimately slower and less-precise, reliance on calculating the short-term sample mean employed by the weighted-average method (Fig. 4a). A potential disadvantage of this recall method is that it requires previously learning the statistics of the environments, but the ultimate outcome—more rapid and precise adaptation to previously experienced environments—has obvious benefits for listening performance. We propose, therefore, that on first encountering a novel sound environment, the auditory brain exploits a mechanism that estimates the short-term sample mean and that this reflexive, but slow, adjustment of neural firing rates is implemented by, or at, the level of the midbrain. We further propose that, however, over time the different statistical environments encountered are learned at a cortical level and, via the corticofugal pathway, this learning accelerates adaptation in the midbrain. In this framework, when cortical input is lacking, midbrain neurons revert to a simple averaging of sound intensity with little capacity to adapt more quickly to previously encountered sound environments (Fig. 4c).

## Discussion

We investigated whether adaptation time-scales are fixed and independent aspects of the adaptation process of sensory systems, or are themselves subject to adaptation over time. The time-scale of adaptation in the auditory system—and sensory systems more generally—has been the subject of increasing research interest as the crucial role of adaptation in neural coding and sensory processing has become apparent. We have demonstrated that functional adaptation to stimulus statistics, which improves neural coding of sound level in the auditory midbrain, accelerates each time an environment is re-encountered. This meta-adaptation is mediated by the corticofugal system, and constitutes a novel form of sensory memory, enabling increasingly repeated environments to be encoded more precisely with increasing speed.

Our data—consistent with previous findings that adaptation in the IC occurs on the order of hundreds of milliseconds to changes in the mean sound intensity, as well as to higher-order statistics such as the variance and kurtosis,—suggest two new and surprising features of the time course of adaptation. First, adaptation time-scales appear not to be fixed and invariant but are, rather, themselves subject to an adaptive process over time, such that adaptation speeds up with repeated stimulus presentations. This acceleration of adaptation results in a more rapid improvement in neural coding of sound levels as the brain accrues past experience of the sounds. This longer-term change, which occurs on the order of tens of seconds, may be related to the studies of Ulanvosky *et al*.^13^, who showed that, in auditory cortex, several adaptive processes occur concurrently, identifiable by different time-scales, encompassing hundreds of milliseconds and tens of seconds, or to the long-term adaptation of Dean *et al*.^1^ in IC. However, unlike these studies, which describe this timescale as a form of spike-rate adaptation itself, we have uncovered a change in the rate of adaptation—adaptation of adaptation itself—a qualitatively different phenomenon which we term meta-adaptation.

Second, we find that acceleration of adaptation in IC neurons is reduced or abolished when auditory cortex is inactivated by cooling, suggesting that meta-adaptation in the auditory midbrain is influenced by feedback from the auditory cortex. These findings suggest a function for the corticofugal pathways in influencing neural coding of the unfolding sensory environment, expanding the temporal sensitivity—the neural memory—of midbrain neurons, and rendering them sensitive to stimulus repetitions across tens of seconds. This function complements an increasing number of functions recently suggested for this seemingly important pathway including, for example, the re-learning of auditory sensory information following altered sensory input, or in other sensory systems, changes to the representation of visual stimuli^22^,^24^. These findings also suggest a more complex view of the auditory midbrain than is often imagined, as a site where neurons integrate short-term stimulus data from ascending inputs with longer-term sound history provided by ‘top–down' influence from auditory cortex.

That meta-adaptation occurs in the midbrain, and that it is influenced by the activity of the auditory cortex—a processing stage in which sensory integration appears to unfold over an appropriate timescale—raises the question as to what stimulus features (or response features) are responsible for eliciting it. Is it the number of presentations of the quiet, or the loud environment, or the number of transitions between the two, or the cumulative number of evoked action potentials, or—perhaps most consistent with our model hypotheses—the total duration of time (or number of 50 ms epochs) for which the loud environment is experienced? We also cannot say for certain what is the role of meta-adaptation in neural coding, although we speculate with our model that it might be a mechanism to ensure more rapid adjustment to an efficient and precise neural code for the current sound environment by recalling the environment's statistics from previous encounters. It is possible that some environments might be easier to recognize and recall than others, and therefore might evoke meta-adaptation more quickly, or to a greater degree, hence providing a test of the model. Distinguishing two environments with less-overlapping sound-intensity distributions than we employ here, for example, might generate meta-adaptation more rapidly and powerfully, as it would be easier to determine from hearing the intensities of a few epochs which environment was currently experienced. These and several other questions will form the subject of future research into the nature of meta-adaptation.

These findings provide an experimental basis by which to motivate models of neural function in changing environments, and have implications for neural diseases, such as tinnitus, autism and schizophrenia, that involve ‘top–down' processing, aberrant auditory experience and estimation of the changing statistics of an environment^34^,^35^,^36^. We speculate that, as with adaptation, meta-adaptation may be ubiquitous across sensory systems and brain centres.

## Methods

### Sound stimuli and neural recordings

All experiments were carried out in accordance with the Animal (Scientific Procedures) Act of 1986 of Great Britain and Northern Ireland. All procedures were reviewed and approved under UK Home Office Licence (covered by both Project and Personal licenses). Young adult pigmented guinea pigs (*Cavia porcellus*; weight 350–550 g) of both sexes were anaesthetized with urethane and placed in closed-field auditory apparatus as previously described^2^. Stereotaxic craniotomies were performed over the right IC and the auditory cortices bilaterally. Sounds were generated digitally, converted to analogue signals, attenuated and amplified before being presented to within a few millimetres of the tympanic membranes via loudspeaker units. Extracellular recordings were made from single neurons in right IC and AC, using glass-coated tungsten electrodes. Sound-responsive neurons were first detected by presenting broadband noise or tones whilst advancing a single electrode through the IC, until a single neural unit could be isolated. All neurons responsive to broadband noise were presented with the switching stimulus.

Subsequent to isolation, the frequency tuning and other basic aspects of the neural response were characterized using our standard methods^1^. After that the response was recorded to a ‘switching stimulus'^1^. This consisted of a continuous broadband noise stimulus (50–25 kHz bandwidth) presented for 10–30 min, in which the sound intensity was adjusted every 50 ms (an epoch) to a new value chosen randomly from one of two defined distributions. The distributions were discretized in 2 dB steps, spanning from 21 to 85 dB SPL. Each distribution contained a region of highly probable levels (the stimulus ‘high-probability region') over either 51±6 dB SPL (‘quiet') or 75±6 dB SPL (‘loud'), from which levels were selected with an overall probability of 0.8. The two sound level distributions were alternated in time, such that sound intensities were chosen from one distribution for 5 s before switching to the other. Thus each switch period consists of a half-period long presentation of the quiet environment, followed by a half-period long presentation of the loud environment. At least 24 switch periods were played (lasting 4 min), precisely how many depending on how long the neuron could be isolated for. The switching stimulus was played in one of three ways: when recording from a neuron for which no cooling was performed, it was played once (26 neurons); when cooling was performed it was either played continuously before and throughout the cooling process (23 neurons), or it was stopped before cooling and then restarted when the cortex was cooled (29 neurons). The level sequence differed in each switch period (Fig. 1), and for the whole switching stimulus there were two different overall intensity-sequences (‘seeds'), a neuron being presented with just one seed or the other. The interval between recordings from individual neurons, during which time further neurons were being sought, was 5–20 min. Short bursts of broadband noise (50 ms duration) were presented during this ‘search' phase.

### Cortical inactivation by cooling

We modified a proprietary cryoloop cooling system previously described^26^ to accommodate two 4 mm diameter cryoloops which could be placed into craniotomies and apposed to auditory cortices bilaterally. Bilateral cooling avoids the limitations of many studies of the corticofugal pathway that tend to leave the contralateral pathway intact, with the likelihood of incomplete removal of descending inputs. Cooled methanol was passed through the cryoloops and pump speed controlled manually to achieve desired temperatures. To assess the effect of cortical inactivation on midbrain adaptation one of two protocols were performed as mentioned above. For some neurons (23 neurons), ∼10 min of adaptation stimulus was presented, then cortical cooling was rapidly induced and maintained for 10 min once steady-state had been reached. Cooling was then discontinued, and in some cases recordings continued for a further 10 min once cortex had returned to pre-cooled temperatures. For other neurons (29 neurons), to control for possible long-term effects of presenting continuous sound stimuli, a discontinuous stimulus protocol was used, in which stimuli were not presented during the cooling or rewarming transitions, but only once steady states had been reached in each of the warm, cool and re-warm states.

Before these experiments, we conducted extensive calibration and control experiments including recording temperatures at multiple cortical and subcortical depths to ascertain the spread of cooling through the brain, as well as recording in IC whilst cooling non-auditory cortex overlying IC, to ensure that our data could not be explained by a direct temperature effect on IC. Drawing on previous reported cryoloop experiments in the same species and using the same cryoloop design^23^ to ensure appropriate cyroloop dimensions and correct placement on auditory cortex, we performed our calibration experiments in several individual animals. These calibration data are based on the previous finding^26^,^27^ that inactivation of full-thickness cortex with a cryoloop occurs when cortical temperature reaches 20 °C. We therefore measured cortex temperature at a variety of depths from the surface for different cryoloop temperatures, and defined the (surface) loop temperature necessary to achieve a specific temperature 2 mm below the cortical surface (that is, roughly the full thickness of cortex in this species) of 20 °C. The temperature change at a depth of 2 mm when loop temperature is 15 °C, demonstrates that full thickness cortex (2 mm deep) is cooled to 20 °C when surface loop temperature is 15 °C (Supplementary Fig. 3b).

Following this calibration procedure, we then confirmed cortical inactivation for this temperature using multi-unit recordings via electrodes placed stereotactically down through auditory cortex. Neuronal activity recorded at a depth of 2,100 μm below the surface of auditory cortex, that is, at the deepest extent of layer VI, typically ceased entirely after ∼5 min of cooling (Supplementary Fig. 3c). Five minutes after the pump controlling the flow of coolant is switched off, neuronal activity returns. However at 2400 μm below the surface, several hundred micrometers deeper than then deepest extent of cortex, neuronal activity does not cease completely even after cooling for more than 10 min, suggesting that cooling the surface of the cortex to 10 °C is sufficient to cool full-thickness cortex but does not inactivate neurons even a small distance below the deepest levels of the cortex (example neuron, Supplementary Fig. 3d). This was further tested by cooling the loop to 5 °C and recording from the same neuron. At this lower temperature, the neuron was indeed inactivated, suggesting that whilst cooling the cortical surface to 10 °C does not inactivate subcortical structures, 5 °C at the cortical surface is sufficiently low to do so. These measurements were obtained from an animal weighing 510 g (weights of guinea pigs employed in these experiments ranged from 350–700 g). Similar findings were made in several electrode tracks within the AC in three different animals. Having established the principle that, in accord with previously published data, cooling full thickness cortex to 20 °C or lower was sufficient for neuronal inactivation, we did not record AC neuronal activity routinely in every experiment, but did record loop temperature.

### Data analysis

For each neuron we constructed peri-stimulus-time-histograms (PSTHs) for each consecutive presentation of the ‘loud' environment, using time bin size of 50 ms, matching the epochs of the stimulus (Fig. 1). As we needed to measure responses in short time windows given limited data, we then averaged over all the neurons to provide the population PSTH for each consecutive presentation of the loud environment. Then we averaged the population PSTHs over presentations 11–24 (Fig. 2a). We also averaged over the population PSTHs for sets of three consecutive 5 s presentations of the loud environment, to produce a series of PSTHs as a function of presentation number, where the presentation number is the first presentation of the set of three (Fig. 2b,c). Single exponential decay constants were fit to these PSTHs over the 2.5 s following the switch, giving a time-constant *τ*_adapt_ of the change in firing rate (Fig. 2a,b). The fit was performed by minimizing mean squared error over time bins *t* (50 ms bins), between the estimated firing rate *ŷ*(*t*)=*a*+*b* exp(-*t*/*τ*_adapt_) and the true firing rate *y*(*t*), using minFunc (ref. 37). The parameters *a*, *b* and *τ*_adapt_ were randomly reinitialized and fit 100 times and the best fit taken. Goodness-of-fit was assessed against a similarly fitted flat line *ŷ*(*t*)=*a* using the *f*-test. Time-constants of adaptation were thus derived for successive presentations of the loud environments (Fig. 2c). This analysis provided a series of time constants and was the first stage of the analyses for the full data set (78 neurons), the first-recording-excluded data set (64 neurons), the no-single-neuron-fit data set (66 neurons), the cooled data set (52 neurons) and the started-while-cooled data set (29 neurons). As an aside, note that the longer time-constants in the time-constant succession, over the range from presentation 11 to 24 (Fig. 2c), are similar to the time-constant found using all data from switches 11 to 24 (Fig. 2a). This suggests that the longer time-constants (Fig. 2c) dominate in determining the many-presentation-averaged time-constant (Fig. 2a).

To assess how the time course of adaptation might itself change over successive presentations of the loud environment, an exponential decay curve 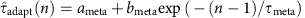 was fit (again using mean squared error, minFunc (ref. 37), and reinitialization 100 times) to the adaptation time-constant *τ*_adapt_(*n*) versus presentation number *n* relationship, with subscript ‘meta' denoting that this is meta-adaptation. Only the first and then every third presentation set was used in the fitting to avoid using overlapping data. Goodness-of-fit was again assessed against a similarly fitted flat line 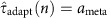 using the *f*-test. Hence this provides a curve describing the meta-adaptation (Fig. 2c) from which the initial time-constant (*a*_meta_+*b*_meta_) and steady-state time-constant (*a*_meta_) could be taken. Measuring adaptation and meta-adaptation in single neurons was performed in exactly the same way as for the population response, but using the PSTHs of single neurons, rather than the PSTHs averaged over the neural population (Supplementary Fig. 1).

For the neural population response, the above procedure was also bootstrapped by selecting a set of neurons with replacement from the population and fitting their adaptation and then meta-adaptation, and repeating this 10,000 times (1,000 for the 64 neuron analysis). The bootstrapping provided a distribution of possible values of the initial and steady-state time-constants of the meta-adaptation for which confidence intervals could then be assessed. This bootstrapping was performed for all neurons for which responses had been recorded to the stimulus (Fig. 2d, 78 neurons). For this analysis in the cooled condition, as the exponential fit was not significant versus a flat line for the case where the stimulus was started in the cooled state (Supplementary Fig. 5, 29 neurons), the adaptation time-constant for the cooled state was assessed as the single number provided from the flat line fit, and included flat line fits (over 24 presentations) for neurons (23 neurons) under the cooled condition where the switching stimulus was played continuously during cooling (Fig. 3a, 52 neurons).

Rate-intensity functions for individual neurons (Fig. 1e, Supplementary Fig. 2) were constructed by finding the average response (Fig. 1d) for a given sound level (Fig. 1b) for either the loud environment presentations or the quiet environment presentations. Rate-intensity functions for the neural population response are the average of all the single-neuron rate-intensity functions in the population. Rate-intensity functions for the neural population response and for single neurons were measured for the loud environment in different states of adaptation and meta-adaptation (Fig. 2e,f, Supplementary Fig. 2). In each case the population rate-intensity function was measured as the adaptation progressed by using data from consecutive spans over the duration of the presentations. Rate-intensity functions were measured using just data from either the first three presentations (Fig. 2e, Supplementary Fig. 2a,c,e) or presentations 10–24 (Fig. 2f, Supplementary Fig. 2b,d,f). Most of the rate-intensity functions were only calculated over the high-probability region as data were limited particularly for the early spans over the presentations. These rate-intensity functions were displayed after a 5-point moving average (which was not used in the calculations in the next paragraph). The variance on population rate-intensity function (Fig. 2f) was calculated separately for each seed, and then the average taken weighted by the relative occurrence of the seeds. The standard deviation was then calculated as the square root of this variance. Then a five point moving average was applied for display.

Measuring the relative discrimination capacity of the neural population rate-intensity function, as a function of adaptation state, and for different states of meta-adaptation, could be done over the high-probability region of the stimulus, as in this region there was sufficient data. Assuming that the overall spike rate represents the current sound level, a relative measure of how well the neural population could discriminate nearby sound levels was calculated (Figs 2g and 3b). The formula^28^,^29^,^30^,^31^,^32^ for this measure (*d′*), was 
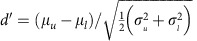
. Here *μ*_u_ is the spike rate averaged over the upper three sound-levels of the high-probability region and *μ*_l_ is the spike rate averaged over the lower three sound-levels of the high-probability region. For steady-state rate-intensity function in the meta-adapted state, there is enough data to calculate the s.d. on the spike rate (Fig. 2f), and it is approximately proportional to the square root of the spike rate. Thus for all our calculations of *d′*, which we only use as a relative measure, we set 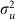 to *μ*_u_, and 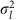 to *μ*_l_. *d′* was calculated for consecutive 400 ms spans over the duration of the switch period, using data from the first three switch periods (Figs 2g and 3b, red lines), switch periods 10–24 (Figs 2g and 3b, black lines) or switch periods 1–24 during the cooled state (Fig. 3b, cyan line). When comparing *d′* in the cool state (Fig. 3b, cyan line, 52 neurons) with *d′* in the warm state (Fig. 3b, red and black lines, 78 neurons), *μ*_u_ and *μ*_l_ were appropriately rescaled to account for the differing neural population sizes. Restricting the warm state *d′* analysis to the 52 neurons that were used for the cool state *d′* analysis also produced qualitatively similar results.

### Modelling

The weighted-average method estimates the fixed mean sound intensity *I*_L_ of the loud environment using a weighted sample of recent sound intensities. This estimate 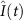 at time epoch *t* is provided by a simple convolution 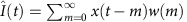. Here integer *t* indicates the 50 ms epoch, where *t*=0 is the first epoch of the loud environment presentation, and thus negative *t* will be the quiet environment and 0 and positive *t* the loud environment, and *x*(*t*) is the sound intensity in dB at epoch *t*. The sampling window *w*(*m*) has the form *w*(*m*)=exp(−*m*/*τ*)/*Z*, where 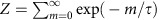 is the normalizing denominator, and *τ* is the time-constant of decay (in epochs) set to match values in the physiology. The RMS error (Fig. 4a, dotted and dashed lines) of the estimate of the mean 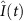 with respect to the true mean *I*_*L*_ of the loud environment, is given by 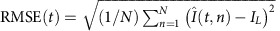, where *n* denotes different switch periods of the loud environment (*N*=1,000 switch periods were used in the models).

The recall method is modelled using a hidden Markov model. The hidden Markov model has two states; the state in a 50 ms epoch indicates whether it believes it is in a loud or quiet environment. The sound levels decompose into just three relevant observations; whether the observed sound level lies within high-probability regions of the loud or quiet distributions or elsewhere. The model (Fig. 4b) ignores long-term time structure of the switching stimulus and assumes state transitions follow a Bernoulli process with a mean transition interval of 100 epochs. From previous experience of the switching stimulus, the model is taken to have learned the appropriate transition probabilities and output probabilities to describe the switching stimulus (shown in Fig. 4b), as well as the mean intensity of the loud environment and of the quiet environment. Given these probability values of the model, and the recent sound level observations, the model can estimate, at time *t* since the start of a presentation, the probability *p*_L_(*t*) of being in the loud environment and *p*_Q_(*t*)=1-*p*_L_(*t*) of being in the quiet environment (the posterior state probabilities)^38^. The estimate of the mean sound level at time *t* is then given by 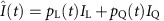, where *I*_L_ and *I*_Q_ are the mean intensity of loud and quiet environment, values memorized from experience. The RMS error of this estimate of the mean (Fig. 4a, solid line) is then calculated as for the exponential averaging model.

### Data availability

All neural response and cryoloop temperature data which support this study are available from the corresponding author upon request.

## Additional information

**How to cite this article:** Robinson, B. L. *et al*. Meta-adaptation in the auditory midbrain under cortical influence. *Nat. Commun.* **7**, 13442 doi: 10.1038/ncomms13442 (2016).

**Publisher's note**: Springer Nature remains neutral with regard to jurisdictional claims in published maps and institutional affiliations.

## Supplementary Material

Supplementary Information Supplementary Figures 1 - 5
