# Supplementary material for: Meta-adaptation in the auditory midbrain under cortical influence
Source: Nat Commun. 2016 Nov 24;7:13442. doi: 10.1038/ncomms13442 (PMC5123015; doi:10.1038/ncomms13442)
Supplement: Supplementary Information — Supplementary Figures 1 - 5 [file ncomms13442-s1.pdf]

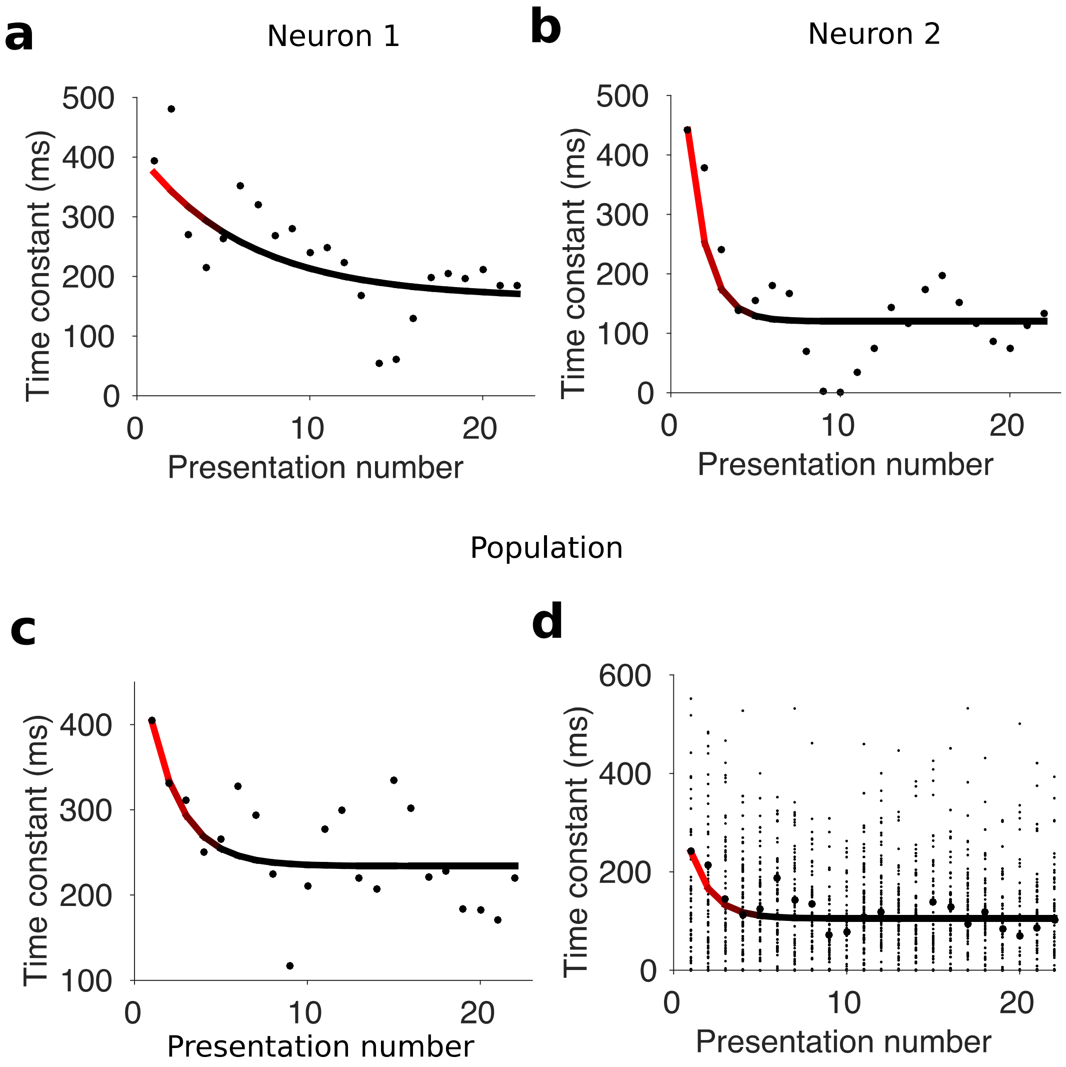

Figure S1 | Meta-adaptation of single units in the auditory midbrain. (**a-b**) Adaptation time constant vs. presentation number for two example neurons. Exponential fit (line). (a) τ_{meta}=6.2. (b) τ_{meta}=1.1. (c) Adaptation time constant vs. presentation number for the neural population with only those neurons (n=66) for which statistically significant meta-adaptation cannot be measured in their single neuron response. (d) Adaptation time constants for every neuron in neural population (n=78) as function of presentation number (small dots). The mean adaptation time constant over the 78 neurons (large dots) shows a significant exponential fit (line) with τ_{meta}=1.2.

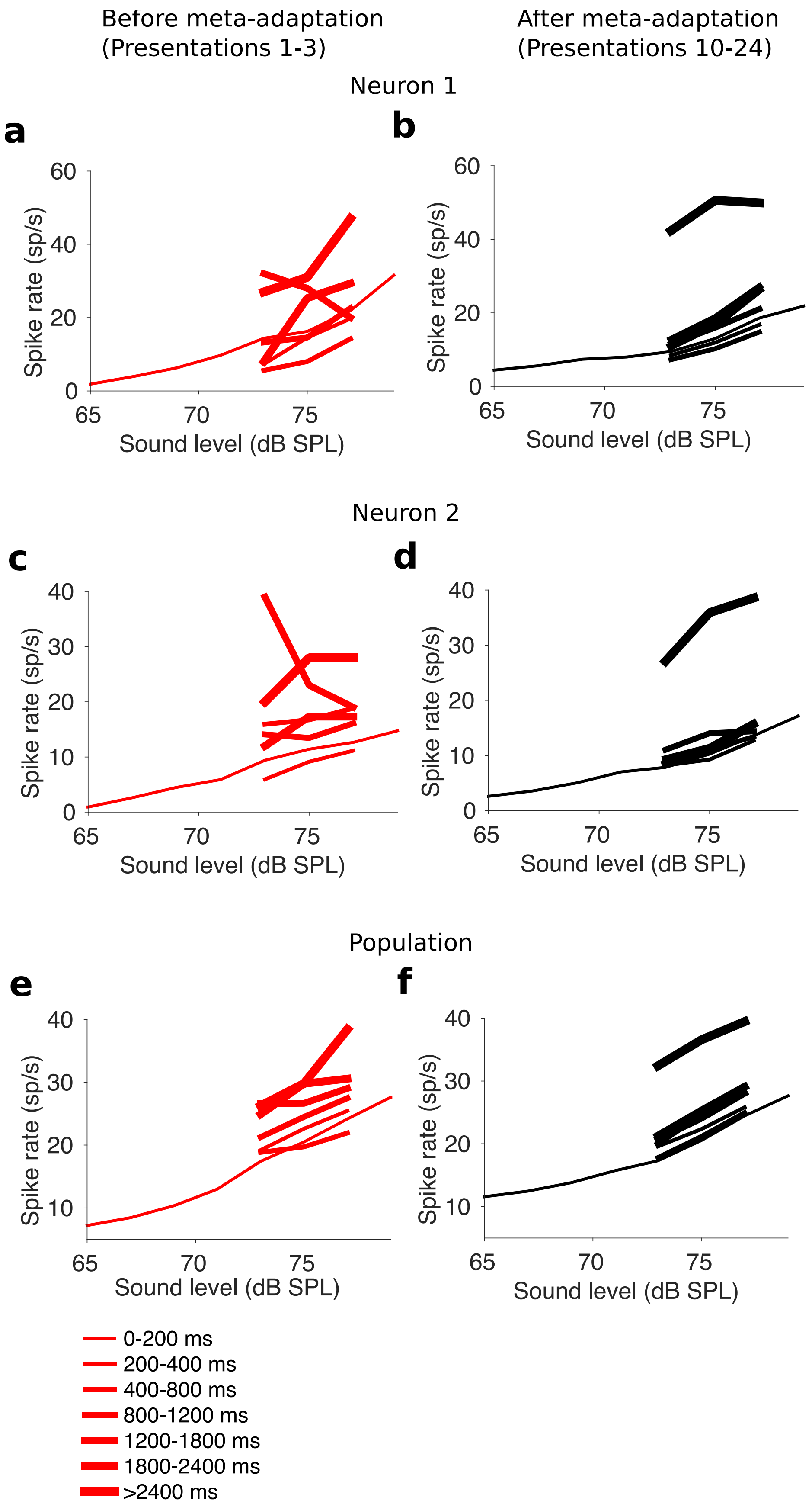
Before meta-adaptation
(Presentations 1-3)
After meta-adaptation
(Presentations 10-24)
Neuron 1
a
b
Neuron 2
c
d
Population
e
f
Spike rate (sp/s)
Sound level (dB SPL)
0
10
20
30
40
60
65
70
75
0-200 ms
200-400 ms
400-800 ms
800-1200 ms
1200-1800 ms
1800-2400 ms
>2400 ms

Figure S2

Figure S2 | Meta-adaptation of rate-intensity functions of single units in the auditory midbrain. (**a-d**) Rate-intensity functions for the high-probability sound levels at times from environment onset, for single neurons. (a) Before meta-adaptation and (b) after meta-adaptation for the neuron in Fig. S1a. (c) Before meta-adaptation and (d) after meta-adaptation for the neuron in Fig. S1b. (**e-f**) Rate-intensity functions for the high-probability sound levels at times from environment onset, for the neural population with only those neurons (n=66) for which statistically significant meta-adaptation cannot be measured in their single neuron response. (e) Before meta-adaptation and (f) after meta-adaptation.

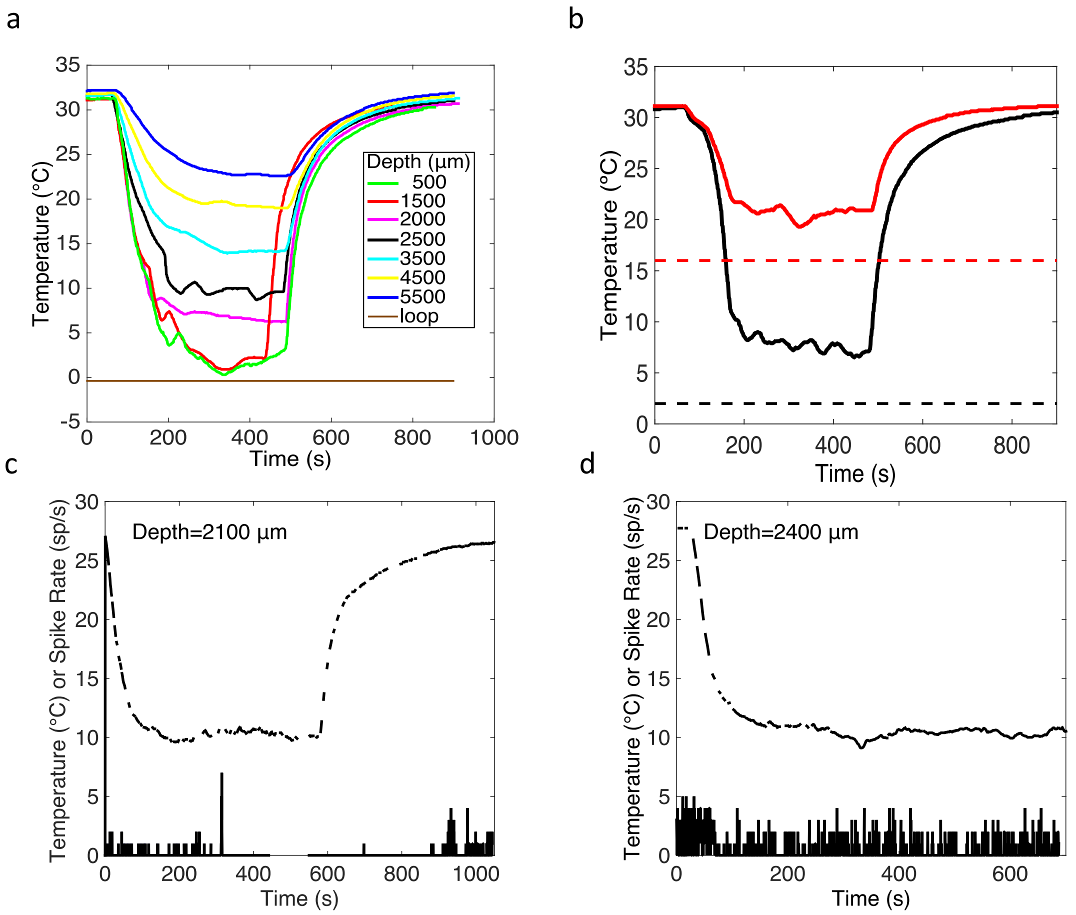

Figure S3 | Cryoloop calibration and cortical inactivation. (a) Temperature at depths below surface of auditory cortex when the cryoloop was held at 0 °C, a temperature commonly employed in other studies. Legend displays depth beneath brain surface. Cortex is 2000 µm thick; a loop temperature of 0 °C leads to the neuronal inactivation temperature of <20 °C extending below cortex (>2000 µm). (b) However, cooling cryoloop to 15 °C was sufficient to cool cortex to the inactivation temperature of 20 °C without appreciably cooling sub-cortex, so a cryoloop temperature of 10-15 °C was selected as sufficient and not excessive for our experiments. (c) Cortical neuronal responses displayed as peri-stimulus time histograms (PSTHs; bin width = 50 ms). A single neurone at a depth of 2100 µm is inactivated by cooling loop to 10 °C. (d) In the same electrode track, a neuron at a depth of 2400 µm, just beneath auditory cortex, is not inactivated by cooling to 10 °C. Temperature and spike count plotted on ordinate to same scale.

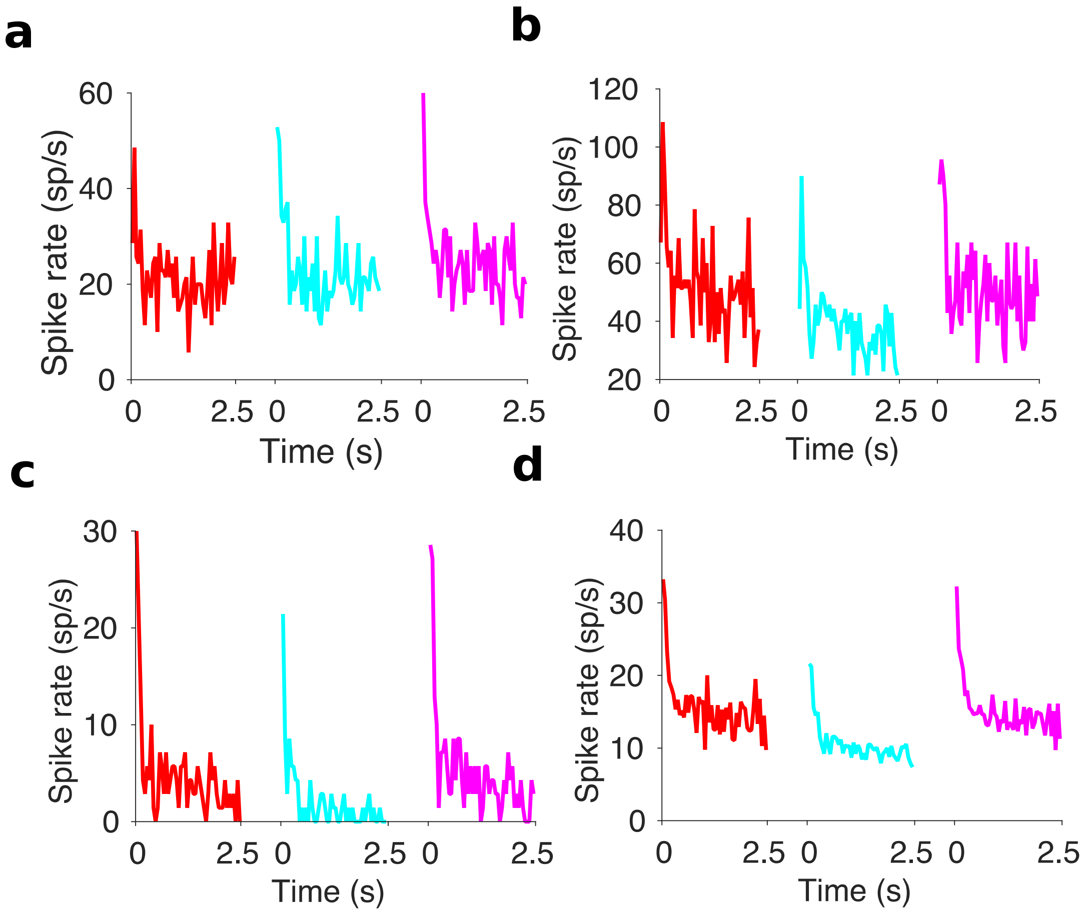

Figure S4 | Neural responses in the midbrain before, during, and after cooling of auditory cortex. (a-c) Single neuron PSTHs for the first 2.5 s of the loud environment of the switching stimulus, averaged over presentations 11-24 from the onset of the stimulus (or from the point where the cortex is cooled or rewarmed if the stimulus is played continuously). Red line, before cooling, cyan line, during cooling, magenta line, after cooling. (d) Population average PSTH for the 26 neurons for which recordings in response to the switching stimulus were made before, during, and after cooling which met recovery criterion, i.e. that rate-level functions in these neurons, which underwent a rightward shift during cortical cooling, shifted leftward again to overlie or lie close to the original rate-level-function curves when cortex was re-warmed. Across the population of these neurons, there was no significant difference between the time-constants of adaptation in the warm and rewarm conditions, implying that these neurons had indeed recovered their original responsivity (mean (warm, cool, rewarm) = 157; 326; 176 ms; t-test warm vs. rewarm, p = 0.665; t-test warm vs. cool, p = 0.009).

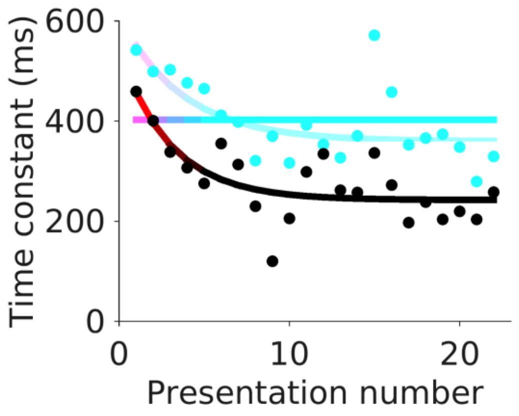

Figure S5 | Adaptation time constant vs. presentation number for 29 neurons in the warm condition. Exponential fit (red-black line). Adaptation time constant vs. presentation number for the same 29 neurons in the cool condition. Flat line fit (pink-blue line). Non-significant exponential fit (pale pink-blue line).
